# Graphene Quantum Dots for Glioblastoma Treatment and Detection–Systematic Review

**DOI:** 10.3390/molecules30122483

**Published:** 2025-06-06

**Authors:** Kacper Kregielewski, Wiktoria Fraczek, Marta Grodzik

**Affiliations:** Department of Nanobiotechnology, Institute of Biology, Warsaw University of Life Sciences, 02-787 Warsaw, Poland

**Keywords:** graphene quantum dots, GQD, glioblastoma, PTT, pretreatment, neoadjuvant therapy, biosensors, bioimaging, drug delivery

## Abstract

Glioblastoma, a highly malignant tumor, has a poor prognosis, necessitating the development of effective therapeutic strategies due to the low success rates of existing treatments. Graphene quantum dots (GQDs) have garnered attention for their unique physicochemical, electronic, and optical properties, along with biocompatibility and the ability to cross the blood–brain barrier. This systematic review evaluates the current applications of GQDs in glioblastoma management. A search across databases such as PubMed, Science Direct, and Web of Science identified 658 papers, with 10 selected for this review based on the eligibility criteria. Most of the selected studies explored GQDs as pretreatment agents for therapies like chemotherapy and photothermal therapy, alongside their roles in biosensing, bioimaging, and drug delivery. Although research is still limited, this review highlights the significant potential of GQDs as multifunctional platforms in glioblastoma therapy. Further studies are essential to optimize these nanostructures for clinical applications, aiming to improve the precision and effectiveness of treatments while reducing systemic side effects.

## 1. Introduction

Nanomedicine is a rapidly evolving interdisciplinary field that utilizes advancements in nanobiotechnology to improve the prevention, diagnosis, and treatment of human diseases [1]. A wide range of nanoparticles have been extensively investigated for their therapeutic potential across diverse biomedical applications, including anticancer interventions, antimicrobial therapies, and angiogenesis modulation [2]. Significantly, nanoparticles have emerged as promising tools for targeted drug delivery due to their capacity to overcome biological barriers, such as the blood–brain barrier (BBB) [3]. Carbon-based nanoparticles are highly promising in nanomedicine due to their exceptional biocompatibility, leading to extensive investigation of their potential applications [4].

One of the prominent carbon-based nanoparticles is graphene quantum dots (GQDs), which have recently garnered significant attention due to their remarkable physiochemical, electronic, and optical properties and biological applications. GQDs are characterized by their outstanding biocompatibility, superior mechanical flexibility, elevated electron mobility, and remarkable thermal and chemical stability [5,6]. GQDs are smaller than 10 nm, consist of one or multiple layers of graphene, and have carbons in sp^2^ hybridization [7,8]. They may be confused with carbon dots (CDs), which have an sp^2^/sp^3^ carbon structure [9]. One of the distinguishing characteristics of graphene quantum dots (GQDs) is their exceptional photoluminescent properties, which are intricately linked to the quantum confinement effect. The emission of light occurs due to the excitation of electrons within a GQD following the absorption of incident photons. This process is further characterized by the subsequent emission of photons as the excited electrons transition back to their original, lower energy state [7,10]. The photoluminescence exhibited by GQDs can be modulated by several factors, including the presence of structural defects and the nature of surface functionalization [11,12,13,14]. Moreover, GQDs’ exhibited photoluminescence is size and pH-dependent [15]. Studies on murine models have demonstrated that GQDs’ administration does not significantly impact vital physiological parameters or organs’ morphological and functional integrity [16]. What is most important in the potential application of GQDs in glioblastoma therapy is the fact that they can cross the BBB, making them a promising approach to its therapy [17].

Glioblastoma is one of the deadliest primary cancers of the brain [18]. The occurrence rate is estimated to be in the range of 3 to 4 per 100,000 individuals [19]. In clinical practice, the standard therapeutic strategy for glioblastoma utilizes radiotherapy, temozolomide (TMZ) chemotherapy, and surgical resection of the tumor [20]. These treatments are associated with significant adverse effects that can negatively affect patients’ overall well-being and quality of life. Currently, implemented glioblastoma therapies do not lead to satisfactory results, as the median overall survival (OS) for glioblastoma patients is approximately 16 months, while in older patients, who are diagnosed more frequently, the OS decreases to about 11 months [21,22]. The BBB represents a major obstacle to the effective delivery of chemotherapeutic agents in glioblastoma, thereby reducing treatment efficacy. Consequently, it is a primary focus of research efforts to enhance drug permeability and improve therapeutic outcomes [23]. The resolution to the challenges posed by the BBB may lie in the development of nanoparticles capable of effectively traversing this barrier, such as gold nanoparticles [24], liposomes [25], nanogels [26], or GQDs [27,28].

A comprehensive understanding of the diverse properties of GQDs will enable researchers to explore their applications in various therapeutic strategies for the treatment of lethal conditions such as glioblastoma. During this manuscript’s preparation, only 10 publications were identified that directly utilized GQDs in research related to glioblastoma applications. This review offers a comprehensive analysis of the applications of GQDs in glioblastoma, emphasizing their roles in photothermal therapy (PTT), neoadjuvant applications, biosensing, and drug delivery systems.

## 2. Results and Discussion

### 2.1. Literature Review Results

**Figure 1:**
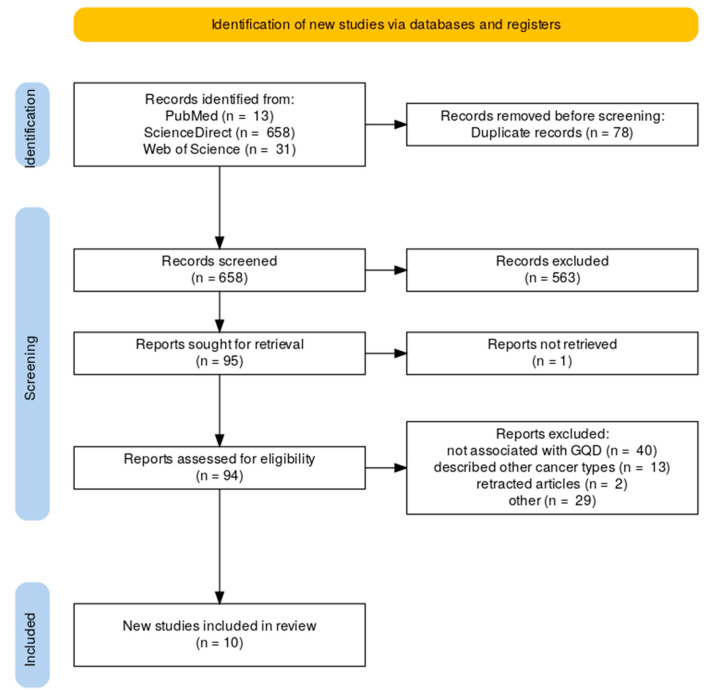
PRISMA flowchart (made using Rayaan).

Following the elimination of duplicates, a total of 658 papers were identified. Subsequent title and abstract analyses led to the identification of 95 articles for comprehensive full-text evaluation. Of these, 85 articles were excluded for the following reasons: the research was not associated with GQD (40 articles), they described cancers other than glioblastoma (13 articles), they were retracted from the publisher’s website (2 articles), and for various other reasons (29 articles). Figure 1 illustrates the flowchart in accordance with the PRISMA statement.

**Figure 2:**
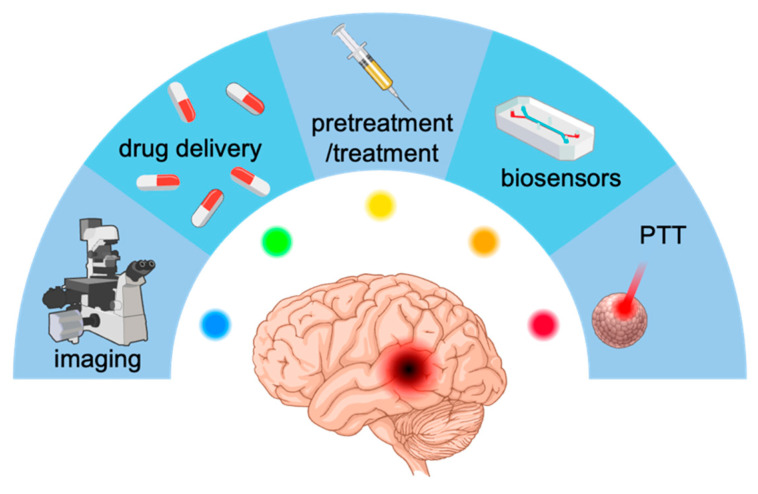
Summary of GQD applications included in the review.

From the 10 selected publications, six different GQD applications, such as photothermal application, neoadjuvant application, biosensor and bioimaging applications, drug delivery and treatment per se, were distinguished, as seen in Figure 2.

A summary of the included studies is presented in Table 1 (GQD properties) and Table 2 (applications).

### 2.2. GQDs as PTT-Inducing Agent

Photothermal therapy (PTT) is an innovative treatment gaining attention in oncology. PTT is often used in conjunction with photodynamic therapy (PDT) and chemotherapy, leading to promising therapeutic outcomes [39,40]. It utilizes PTT agents (PTAs) that convert absorbed light energy into heat when exposed to a specific wavelength of light. This increase in temperature, both around and within the tumor, induces cell death [41]. Cell death as the result of PTT is either achieved through apoptosis, necrosis, or a relatively newly described mechanism called necroptosis. Cell death type in PTT was found to be dependent on the temperature used for the treatment [42]. The primary limitations of PTT are related to the depth of light penetration in biological tissues, risk of imprecise laser exposure and the lack of selective accumulation of photothermal agents (PTA) in non-cancerous tissues. These factors can lead to unintended damage to healthy cells [41,43,44]. Nanoparticle-based PTAs can effectively address the issue of low selectivity in targeting tumors. These nanoparticle-based PTAs utilize nanoparticles’ enhanced permeability and retention (EPR) effect, allowing them to accumulate more effectively in tumor tissue. This characteristic improves their ability to deliver therapeutic agents directly to cancer cells, thus increasing treatment efficacy while minimizing the impact on healthy tissues [44].

Nanoparticles of many types are widely used in research regarding PTT in cancer treatment [45,46]. Examples include the application of gold-based nanoshells as PTAs to enhance the efficacy of liposomal doxorubicin on colorectal tumors in in vivo studies [47], multi-walled carbon tubes and gold nanocomposites for breast cancer PTT [48], or porphyrin immobilized nanographene oxide grafted with l-arginyl-glycyl-l-aspartic-based PTA that resulted in complete elimination of a glioblastoma tumor in an vivo model [49]. For in vivo breast cancer research, GQDs were found to act as a highly biocompatible PTA, successfully inhibiting tumor growth [50].

As for GQD use as a PTT-inducing agent, three publications were identified through the review process. Wang and colleagues successfully synthesized nitrogen and boron-doped graphene quantum dots (N-B-GQD) using 3-aminophenylboronic acid monohydrate, achieving an average size of approximately 4.7 nm. When excited with 808 nm light, the N-B-GQD demonstrated strong fluorescence in the NIR-II window (Near-Infrared II region; 950–1100 nm). Importantly, these synthesized N-B-GQDs were assessed for biocompatibility, revealing no toxicity in both in vitro and in vivo studies. As a photothermal agent, N-B-GQDs effectively inhibited tumor growth when irradiated with an 808 nm laser for 5 min in mice. Furthermore, N-B-GQD emerged as a noteworthy in vivo NIR-II bioimaging agent, facilitating the visualization of internal organs and blood vessels [29].

Wang and colleagues synthesized a nanocomposite utilizing GQDs with similar properties. The GQDs were utilized to create polyamine and graphene quantum dot-capped Prussian blue nanocubes (PB@PDA@GQD). The resulting nanocomposite showed no toxicity toward non-cancerous BV2 cells (a murine microglial cell line). In contrast, cancerous C6 cells demonstrated over 80% survival across all tested concentrations (0–200 μg/mL), indicating the composite’s low toxicity. When treated with an 808 nm laser, C6 cells exposed to 200 μg/mL PB@PDA@GQD experienced a significant decrease in viability, dropping to just 8%. Additionally, the PB@PDA@GQDs induced PTT in mice, inhibiting tumor growth compared to composites that did not incorporate GQDs [32]. The addition of GQDs to Prussian blue and polydopamine, both of which have demonstrated promising applications in cancer PTT [51,52,53], enhanced its PTT capability both in vitro and in vivo [32]. Additionally, it was found that PB@PDA@GQDs were successfully taken up by cancerous cells and exhibited fluorescence while excited with a 405 nm laser, which was not observed in the case of BV2, which suggests additional bioimaging capability in the PTT application [32].

The application of GQDs in PTT for glioblastoma, combined with chemotherapy, was evaluated by Perini and colleagues. They used GQDs with a carboxyl group that measured between 6 and 10 nm in diameter. The study found that GQDs were not toxic to either non-cancerous cells or glioblastoma cells (U87 cells) at concentrations up to 250 μg/mL. However, GQD treatment did increase the membrane fluidity of the glioblastoma cells. When U87 cells were pretreated with 200 or 250 μg/mL of GQDs and then exposed to 1 μM of Dox or 100 μM of TMZ, there was a significant increase in cell mortality compared to cells that were only treated with the same concentrations of Dox or TMZ [36]. Furthermore, Perini and colleagues implemented 3D U87 cultures to explore further GQDs pretreatment and PTT applications. U87 spheroids treated with 200 μg/mL of GQDs for either 7 or 14 days showed no difference in growth or viability compared to the control spheroids. When pretreated spheroids were exposed to either DOX or TMZ, both treatments resulted in an increased toxic effect. No difference in spheroid growth was found between spheroids treated with GQD and treated with either Dox or TMZ for 14 days. Regarding GQD PTT application, it was observed that the GQDs irradiated with an 808 nm laser significantly enhanced the permeability of spheroids. This enhancement was compared to the untreated control group and spheroids that were solely treated with GQD without laser irradiation. In subsequent experiments, spheroids underwent pretreatment with GQD, followed by the administration of either Dox or TMZ. This approach was accompanied by irradiation at a wavelength of 808 nm. The results indicated that the combination of PTT and chemotherapy exhibited a significantly enhanced antitumor efficacy compared to the application of the chemotherapy agent alone or in conjunction with GQD. PTT-enhanced chemotherapy using Dox for spheroids resulted in a significantly greater penetration depth of the antitumor drug compared to Dox alone or treatments involving GQD without 808 nm irradiation.

Additionally, Perini and colleagues found that the production of reactive oxygen species (ROS) and the recruitment of immune cells were notably elevated in spheroids subjected to GQD-mediated PTT [36]. Perini and colleagues also developed the INSIDIA 2.0 (Invasion Spheroid ImageJ Analysis) macro and used it to test the effects of a treatment combining carboxylated GQDs, Dox, and PTT. The results obtained thanks to the INSIDIA 2.0 macro showed that this treatment led to a 70% reduction in the initial volume of spheroids after 14 days [35].

### 2.3. GQDs as a Pretreatment Agent in Neoadjuvant Glioblastoma Therapy

Neoadjuvant therapy is defined as any treatment administered before the primary intervention for cancer, aiming to enhance its effectiveness [54,55]. This may include modalities such as chemotherapy, radiation therapy, and hormone therapy [55]. Within the context of this review, pretreatment is conceptualized as a form of neoadjuvant therapy that is based on GQD as a chemotherapeutic agent.

In clinical practice, the neoadjuvant approach has been used for years to treat breast cancer [56], pancreatic cancer [57], and colorectal cancer [58]. Nanoparticles have garnered significant attention in research as effective pretreatment agents [59]. For instance, liposome-encapsulated doxorubicin has been employed in the targeted therapy of HER2-overexpressed breast cancer [60]. Furthermore, the utilization of dendritic mesoporous silica nanoparticles, combined with superoxide dismutase and catalase, has been demonstrated to inhibit breast cancer metastasis [61]. Despite numerous neoadjuvant therapies for glioblastoma undergoing preclinical and clinical trials, no FDA-approved treatment options are currently available [62]. Bihan and colleagues demonstrated that the pretreatment of glioblastoma patients with bevacizumab in combination with either TMZ or fotemustine prior to the initiation of radiotherapy is associated with enhanced overall survival outcomes [63]. Graphene oxide has been used previously to pretreat glioblastoma and cervical cancer cells, leading to an increase in the toxicity of cisplatin [64]. Additionally, GQDs have been shown to enhance cisplatin uptake by cells, including those resistant to cisplatin [65,66].

During the review process, four publications were identified that discuss the application of GQD as a pretreatment agent for glioblastoma. Perini and colleagues evaluated the biocompatibility and potential pretreatment applications of non-functionalized GQDs (NF-GQDs) and dimethylformamide-functionalized GQDs (DMF-GQDs) prior to the administration of doxorubicin (Dox). Their findings revealed that both types of GQDs exhibited non-toxic behavior at low concentrations (0–150 μg/mL) for both glioblastoma and non-cancerous cell lines. However, the DMF-GQDs showed toxicity at 200 and 250 μg/mL concentrations for both cell types. When treated with 1 μM of Dox, the NF-GQDs at concentrations of 200 and 250 μg/mL were effective in reducing the viability of U87 cells, while leaving non-cancerous cells unaffected. In contrast, treating cancer cells with DMF-GQDs at concentrations of 100, 200, and 250 μg/mL, combined with 1 μM of Dox, also resulted in decreased viability in the cancer cells, with no impact on healthy cells. Both types of GQDs demonstrated the ability to increase the permeability of cancer cell membranes, subsequently enhancing the uptake of Dox. No impact was observed on cortical neurons. The results indicate that co-administration of doxorubicin after treatment with either DMF-GQDs and NF-GQDs yielded a synergistic cytotoxic effect in U87 glioblastoma cells. Specifically, DMF-GQDs enhanced doxorubicin efficacy across all concentrations tested, whereas NF-GQDs demonstrated synergy selectively at concentrations of 200 and 250 μg/mL. The potential clinical application is significant. When DMF-GQD was administered at a non-toxic concentration of 50 μg/mL, followed by 1 μM of Dox, cell viability was reduced to a level that corresponds with doxorubicin’s IC50 of 2 μM (as reported by the authors).

Additionally, administering DMF-GQDs at a non-toxic concentration of 100 µg/mL further decreased cell viability to levels significantly lower than the IC50 observed with doxorubicin alone [30]. These results indicate that administration of DMF-GQDs at non-toxic concentrations (<200 μg/mL) may allow for a reduction in the required dose of doxorubicin in chemotherapeutic regimens. This strategy could reduce dose-dependent adverse effects, notably cardiotoxicity, while preserving or enhancing the treatment’s antitumor efficacy [30,67]. In another study by Perini and colleagues, various types of GQDs were investigated for their potential as a pretreatment in combination with Dox. They comprehensively assessed the impact of these GQDs on cellular functions, including the induction of ROS production, DNA fragmentation, and alterations in cytokine profiles. Three types of GQDs were used: GQDs without surface-specific functionalization (Green GQD), carboxylated GQDs (COOH-GQD), and aminated GQDs (NH2-GQD). All have been found to be smaller than 10 nm in diameter. From all tested GQD types, only Green GQD at the concentration of 250 μg/mL caused a mild reduction in the viability of U87 cells. In contrast, no viability reduction was detected at any examined concentrations (0, 50, 100, 200, 250 μg/mL) for cortical neurons. It was found that all tested GQDs, regardless of used concentrations, did not cause increased ROS production, DNA fragmentation, or an increase in cytokines production (TNF-α, IL-6 and IL-10 for U87 and TNF-α, IL-6 and BDNF for cortical neurons). Administration of 1 μM doxorubicin that followed pretreatment with different types of GQDs resulted in a significant decrease in U87 cell viability only when Green GQDs were applied at 250 μg/mL and COOH- GQDs were used at both 200 and 250 μg/mL. NH_2_-GQDs did not enhance Dox cytotoxicity. Significantly, none of the GQD formulations, at any concentration tested, negatively affected the viability of cortical neuron cells. The administration of 1 μM Dox, following pretreatment with GQDs, resulted in a significant decrease in U87 cell viability only with Green GQDs at a concentration of 250 μg/mL and COOH-GQDs at both 200 and 250 μg/mL, while NH_2_-GQDs did not enhance the cytotoxic effects of doxorubicin. Notably, none of the GQDs tested, at any concentration, negatively impacted the viability of cortical neuron cells. Green and COOH-GQDs significantly enhanced doxorubicin uptake and increased membrane fluidity in U87 cells without affecting cortical neurons. In contrast, NH_2_-functionalized GQDs did not alter doxorubicin uptake or membrane fluidity in either U87 cells or cortical neurons. These results suggest that the biological properties of GQDs are cell type-specific and depend on surface chemistry. Green- and COOH-functionalized GQDs significantly improved membrane permeability and Dox uptake in U87 glioblastoma cells, reducing viability and underscoring their potential use as a pretreatment. On the other hand, NH2-GQDs were determined to be inappropriate for these types of applications [31].

Recognizing that the biological properties of GQDs are dictated by surface chemistry, Perini and colleagues extended their investigation to more biologically relevant models such as neurospheres. Carboxylated GQDs (COOH-GQD), aminated GQDs (NH2-GQD), and unfunctionalized (GQD) were used. A biocompatibility assay showed that even after treatment for 14 days at all tested concentrations (50, 100, 200 μg/mL), all tested GQDs were not toxic and did not cause a significant reduction in U87 neurospheres’ viability. In neurospheres treated with 200 μg/mL of GQDs, unfunctionalized GQDs and COOH-GQDs induced notable growth inhibition. Additionally, these treatments resulted in a significant decrease in membrane fluidity, disruption of cellular clustering, and impairment of intercellular connections. No such effects were found in neurospheres incubated with 200 μg/mL of NH2-GQDs. The results above demonstrate that the surface chemistry of GQDs influences the biology of glioblastoma cells not only in two-dimensional in vitro cultures but also in more biologically relevant three-dimensional culture systems. These findings suggest that COOH-functionalized GQDs and unmodified GQDs can modulate tumorigenesis, offering a promising approach for the therapeutic management of glioblastoma, and should be further studied [33].

### 2.4. GQDs as a Component of Glioblastoma Detecting Biosensors

A biosensor is a device that detects biological or chemical reactions by producing signals proportional to an analyte’s concentration. These devices find applications across various fields. In the biomedical sector, biosensors are utilized for drug discovery and disease detection [68]. A biosensor consists of three key components: a bioreceptor layer with immobilized biomaterials, a transducer that converts biological responses into electric signals, and an electronic system for amplifying and recording these signals for interpretation [69]. A diverse array of biological entities can be utilized in the biological component of biosensors. These entities may include enzymes, antibodies, nucleic acids, proteins, and cellular structures or tissues [70]. The transducer is critical in converting biological signals into physiochemical signals and offers a range of detection modalities. These modalities include electrochemiluminescence, optical detection, electrochemical measurement, fluorescence, and piezoelectric methods [71]. GQDs have been extensively studied as potential components in biosensor assemblies, particularly as electrode surfaces [72]. GQDs exhibit properties such as photoluminescence, a large surface area, broad functionalization capability, high electrical conductivity, and electrochemical sensitivity. They also demonstrate biocompatibility and low toxicity [73]. GQDs have been used as a component of biosensors for cancer detection [74,75,76] and heart diseases, such as myocardial infarction [77].

During the review process, we identified two publications that employed GQDs in developing biosensors aimed explicitly at detecting glioblastoma cells. Ganganboina and colleagues synthesized sulfur-doped GQDs (S-GQDs) using citric acid and 3-mercaptopropionic acid. The size of the synthesized S-GQDs ranged from 4 to 11 nm. The S-GQDs were then deposited onto gold nanoparticle-decorated carbon nanospheres (Au-CNS), forming S-GQDs@Au-CNS, which was followed by the conjugation of angiopep-2 (Ang-2), forming Ang-2/S-GQDs@Au-CNS. Ang-2 is a peptide that binds to low-density lipoprotein receptor protein 1 (LRP1) [78,79], which is highly expressed in glioblastoma, making it a significant target for glioblastoma detection [79]. The composite was deposited onto a glass carbon electrode (GCE), creating the final glioblastoma detection biosensor (Ang-2/S-GQDs@Au-CNS||GCE). The developed biosensor is based on the impedance change induced by the attachment of the glioblastoma cells. Owing to their exceptional surface functionalization capabilities, S-GQDs are employed as an effective conjugator for the immobilization of Ang-2. The biosensing procedure was tested by adding either known amounts of glioma cells in 0.1 M PBS or 10% diluted human serum matrix, followed by a 10 min incubation and a gentle wash with deionized water. Glioma cells present in the sample are identified by a measurable decrease in conductivity, which occurs when these cells bind to immobilized Ang-2 on the biosensor’s surface, forming a specific bioconjugate. The fabricated Ang-2/S-GQDs@Au-CNS||GCE biosensor demonstrates a broad linear detection range from 100 to 100,000 cells/mL, with a limit of detection (LOD) as low as 40 cells/mL. Importantly, its analytical performance remain robust following prolonged storage and in the presence of non-target cell populations. These results indicate that this biosensor is a reliable and selective platform for detecting glioma cells in both phosphate-buffered saline (PBS) and human serum matrices [34]. Wang and colleagues also took advantage of the exceptional surface functionalization capabilities of S-GQDs. They employed an S-GQD as an effective conjugator for immobilizing Ang-2, analogous to the approach used in the biosensor made by Ganganboina and colleagues, as described above. The assembled biosensor consisted of Ang-2, an S-GQD and a nanoporous gold (NPG) composite on GCE (Ang-2/GQDs-NPG/GCE). Additionally, they constructed NPG/GCE and GQDs-NPG/GCE biosensors. To detect glioblastoma cells, they used U251 cells and various other cancerous (non-glioblastoma) and non-cancerous cells to evaluate the biosensor’s selectivity. The developed biosensor functions on impedance principles, showing increased impedance upon conjugation with glioblastoma cells, correlated with cell concentration. The detection procedure was tested by adding known concentrations of U251 cells in 10% serum diluted with PBS, followed by a 30 min incubation at 4 °C and then detected by electrochemical impedance spectroscopy (EIS) in [Fe(CN)_6_]^4−/3−^ solution. To assess selectivity, the biosensor previously incubated with U251 cells was then incubated for 30 min at 4 °C with various cell suspensions and detected by EIS. The fabricated Ang-2/GQDs-NPG/GCE biosensor demonstrated selective detection of U251 cells, achieving an LOD of only 1 cell/mL. Moreover, it maintained excellent stability for 21 days post fabrication [38].

Selective detection of glioblastoma cells using Ang-2 in the samples is a promising approach for further biosensor applications. As mentioned before, LRP-1, detected by Ang-2, is highly expressed on the glioblastoma cells surface. Wang and colleagues utilized U251 as a glioblastoma model; however, they included LN229 in the potentially interfering cell line group, which, according to the ATCC, is also classified as a glioblastoma line [80]. The study observed no affinity for the LN229 cells, indicating a potential absence of LRP-1 on the surface. However, contrasting findings from other research have demonstrated the presence of LRP-1 [81]. Additional research or more information should be provided by the authors.

### 2.5. GQDs for Drug Delivery in Glioblastoma

Drug delivery refers to the controlled transport and release of therapeutic agents to specific locations within the human body. This concept is especially crucial in cancer treatment, where targeted delivery is vital. Many anticancer drugs are highly toxic and can lead to severe side effects throughout the body if they are not accurately directed to the tumor site [82,83]. One of the primary challenges in the treatment of brain cancer is the blood–brain barrier. This physiological barrier protects the brain from harmful toxins and pathogens under normal conditions. However, its selective permeability often restricts the passage of therapeutic agents, thereby significantly limiting their effectiveness in clinical interventions for brain tumors [84]. To address this challenge, nanoparticles have emerged as a promising drug delivery vehicle due to their nanoscale size, which enables them to navigate through the BBB and enable targeted therapeutic delivery [3]. Previous research reported that liposomes, nanogels, dendrimers, metallic and polymeric nanoparticles, as well as quantum dots, have been used to deliver drugs through the BBB [27,28]. GQDs have been effectively utilized as drug delivery carriers for cisplatin in treating breast cancer cells [85]. Additionally, they have demonstrated the capability to co-deliver both cisplatin and Dox for the targeted treatment of nasopharyngeal carcinoma [86].

A single publication by Ostovar and colleagues was identified that specifically addresses the application of GQDs in drug delivery for glioblastoma. The authors synthesized GQDs from citric acid, achieving an average size of approximately 7.99 nm. The synthesized GQDs were incorporated into a co-biopolymer matrix composed of chitosan (CS) and carboxymethyl cellulose (CMC) hydrogel, in combination with zinc oxide (ZnO), to enable the delivery of quercetin (QUR) to the cancer cells, resulting in the formation of a CS/CMC/GQD/ZnO@QUR nanocomposite. The study showed that the CS/CMC/GQD/ZnO@QUR nanocomposite significantly improved the cytotoxic effects on U87 glioblastoma cells, resulting in a 13% greater cell viability reduction than QUR alone. Additionally, the nanocomposite displayed only a slight increase in toxicity toward non-cancerous cells, approximately 2% higher than that seen with pure QUR. The authors assumed this level of toxicity to be acceptable for non-cancerous cells [37].

## 3. Methods

### 3.1. Literature Review

This systematic review followed the Preferred Reporting Items for Systematic Reviews and Meta-Analysis (PRISMA) guidelines [87]. Two authors performed a systematically comprehensive literature search of the PubMed, Science Direct, and Web of Science databases. The initial literature review was conducted on 27 January 2024, followed by an updated search on 22 March 2025. An assortment of keyword searches was employed to formulate a search strategy. The keywords encompassed “graphene quantum dots”, “GQD”, “glioblastoma”, “glioma”, and “GBM”, utilized in both AND and OR combinations. Studies were retrieved utilizing the following Boolean operators: (“graphene quantum dots” OR “GQD”) AND (“glioblastoma” OR “glioma” OR “GBM”). A search filter was applied to restrict results to studies published within the designated period of the past 5 years (2019–2024).

Studies were chosen based on the following criteria: they must be published in English and include either in vitro or in vivo research related to GQD and glioma. The criteria for exclusion included editorials, case reports, literature reviews, meta-analyses, book chapters, in silico studies, retracted papers, and research not focused on GQD and glioma.

The compilation of eligible studies was imported into the Rayyan software, (www.rayyan.ai, (accessed on 27 January 2024–22 March 2025)) [88], and duplicate entries were eliminated. Subsequently, two independent investigators evaluated the results in accordance with the specified inclusion and exclusion criteria.

### 3.2. Data Extraction

For each study included in the review, we meticulously extracted the following data: authors, publication year, type of GQD, their application, source of GQD, synthesis method, precursors, nature of experimental use, and size of the GQD. Furthermore, for the in vitro studies, the cell lines employed, the configuration of the assays, and the observed effects were extracted, while for the in vivo studies, the animal model utilized, and the observed effects were documented.

## 4. Conclusions and Perspectives

In conclusion, this review highlights the potential of GQDs in managing glioblastoma. Owing to their exceptional physiochemical, electronic, and optical properties and their proven biocompatibility and ability to traverse the blood–brain barrier—GQDs have emerged as promising nanoplatforms for targeted therapy. The literature indicates that GQDs can be effectively integrated into photothermal therapy, neoadjuvant pretreatment protocols, biosensing devices, and drug delivery systems, thereby enhancing the precision and efficacy of glioblastoma treatment while mitigating systemic toxicity. The cited studies demonstrate that GQDs functionalized with COOH groups exhibit favorable properties for applications in PTT and neoadjuvant pretreatment. The studies included in our review examined GQDs that were doped with nitrogen, boron, sulfur, or they had dimethylformamide, carboxyl or amine functional groups, as well as GQDs without any additional group or elements present. Additionally, there are numerous other possibilities of doping of functionalization that remain unexplored, revealing a significant knowledge gap that presents opportunities for further research. The contributions of the research team led by Perini to the study of GQD in the context of glioblastoma are notable and warrant recognition. Although only a few studies have investigated GQD applications in glioblastoma, their proven properties highlight their potential as effective nanoparticles in glioblastoma therapy, hereby requiring additional research to move into clinical applications to combat glioblastoma.

## Figures and Tables

**Table 1:** Physiochemical properties of GQDs included in the study.

Author	Year	Symbol	Full Name	GQD Source	Size (Method)	Use
Wang et al. [29]	2019	N-B-GQD	Graphene quantum dots doped with nitrogen and boron	Synthesis by authors	4.7 nm (TEM)	PTT/imaging
Perini et al. [30]	2020	NF-GQD	non-functionalized graphene quantum dots	Sigma Aldrich, St. Louis, MO, USA	<10 nm (DLS, AFM)	pretreatment
		DMF-GQD	dimethylformamide-functionalized graphene quantum dots	ACS Materials, Pasadena, CA, USA		
Perini et al. [31]	2020	Green GQD	no surface-specific functionalization	Sigma Aldrich, St. Louis, MO, USA	<10 nm (DLS, AFM)	pretreatment
		COOH-GQD	carboxylated graphene quantum dots	ACS Materials, Pasadena, CA, USA		
		NH2-GQD	aminated graphene quantum dots	ACS Materials, Pasadena, CA, USA		
Wang et al. [32]	2021	PB@PDA@GQD	polydopamine and graphene quantum dot-capped Prussian blue nanocubes	XFNANO Co. Ltd., Nanjing, China	ND	PTT
Perini et al. [33]	2021	COOH-GQD	carboxylated graphene quantum dots	ACS Materials, Pasadena, CA, USA	<10 nm (TEM)	Treatment
		NH2-GQD	aminated graphene quantum dots	ACS Materials, Pasadena, CA, USA		
		GQD	no surface-specific functionalization	Sigma Aldrich, St. Louis, MO, USA		
Ganganboina et al. [34]	2021	S-GQDs@@Au-CNS	sulfur-doped graphene quantum dots@gold-carbon nanosphere	Synthesis by authors	4–11 nm (TEM)	Biosensor
Perini et al. [35]	2022	GQD	carboxylated graphene quantum dots	ACS Materials, Pasadena, CA, USA	>10 nm (DLS, AFM)	Pretreatment
Perini et al. [36]	2023	GQD	carboxylated graphene quantum dots	ACS Materials, Pasadena, CA, USA	~10 nm (DLS, AFM)	PTT/Pretreatment
Ostovar et al. [37]	2023	CS/CMC/GQD/Zn@QUR	Co-biopolymer of chitosan/carbomethyl cellulose hydrogel improved by zinc oxide and graphene quantum dots nanoparticles	Synthesis by authors	7.99 nm (DLS)	Drug delivery
Wang et al. [38]	2025	Ang-2/GQD-NPG/GCE	electrochemical biosensor based on graphene quantum dots-nanoporous gold nanocomposite	Synthesis by authors	~7 nm (TEM, HR-TEM)	Biosensor

**Table 2:** Applications of GQDs included in the study.

Author	Year	Symbol	Use	In Vitro Models (Doses) (Glioblastoma Model Bolded)	In Vivo Models (Doses)	Outcomes
Wang et al. [29]	2019	N-B-GQD	PTT/imaging	**SF763**; 4T1; B16F10 cell lines (12.5; 25; 50; 100; 200; 500 μg/mL)	Nude mice/nude mice with induced C6 glioblastoma (1 mg/mL)	N-B-GQDs were non-toxic at 500 µg/mL over 72 h but reduced glioblastoma cell viability at 100 µg/mL with NIR exposure. In mice, N-B-GQDs combined with NIR effectively inhibited tumor growth and improved visualization of blood vessels and organs, without harming histological or blood parameters.
Perini et al. [30]	2020	NF-GQD	pretreatment	**U87**; cortical neurons from E15-18 C57BL/6 mice (50, 100, 200, 250 μg/mL)	-	NF-GQD is biocompatible at 50–250 µg/mL for both glioblastoma and non-cancerous cells. In U87 cells, NF-GQD (200–250 µg/mL) combined with 1 µM Dox significantly reduced viability and enhanced Dox uptake (maximal at 250 µg/mL).
		DMF-GQD				Conversely, DMF-GQD (200–250 µg/mL) reduced viability in both cell types. Pretreatment with DMF-GQD (100–250 µg/mL) plus 1 µM Dox further decreased viability and increased uptake. Both NF-GQD (200–250 µg/mL) and DMF-GQD (all doses) exhibited synergistic effect with Dox.
Perini et al. [31]	2020	Green GQD	pretreatment	**U87**; cortical neurons from E15-18 C57BL/6 mice(50, 100, 200, 250 μg/mL)	-	Green GQD (250 µg/mL) slightly reduces the viability of U87 cells by approximately 20%, but it does not have a significant effect on reactive oxygen species (ROS), DNA fragmentation, or cytokine levels. When pretreated with GQD and then treated with 1 µM doxorubicin, it significantly decreases U87 cell viability and enhances the uptake of Dox, as well as membrane fluidity, without impacting cortical neurons.
		COOH-GQD				COOH-GQD on its own does not affect cell viability or cytokine levels. However, when administered at concentrations ranging from 200 to 250 µg/mL prior to Dox, it decreases U87 viability while increasing Dox uptake and membrane fluidity, without any effects on cortical neurons.
		NH2-GQD				NH_2_-GQD exhibits no influence on viability, ROS, DNA fragmentation, cytokine levels, Dox uptake, or membrane fluidity in either cell type.
Wang et al. [32]	2021	PB@PDA@GQD	PTT	**C6**, BV2 (12,5; 25; 50; 75; 100; 200 μg/mL)	Balb/c mice with induced C6 glioblastoma (6 mg/kg)	PB@PDA@GQD is non-toxic to BV2 cells and maintains over 80% C6 cell viability across 12.5–200 µg/mL, but 200 µg/mL PB@PDA@GQD with NIR decreases C6 viability to 8%. In Balb/c mice with C6 tumors, PB@PDA@GQD with NIR significantly inhibits tumor growth.
Perini et al. [33]	2021	COOH-GQD	Treatment	**U87MG** (50, 100, 200 μg/mL)	-	COOH-GQD at concentrations of 50, 100, and 200 µg/mL did not result in significant changes in U87 cell viability over a period of 14 days. However, COOH-GQD at 200 µg/mL inhibited neurosphere growth, affecting the average size and density, and also led to a decrease in membrane fluidity while impacting clusterization and connection formation.
		NH2-GQD				NH_2_-GQD at the same concentrations did not affect U87 cell viability after 14 days, did not inhibit neurosphere growth, showed no significant change in membrane fluidity, and did not influence clusterization or connection formation.
		GQD				GQD, similarly to COOH-GQD at concentrations of 50, 100, and 200 µg/mL also showed no significant effect on U87 cell viability. GQD 200 µg/mL inhibited neurosphere growth and caused a decrease in membrane fluidity, while also inhibiting clusterization and connection formation.
Ganganboina et al. [34]	2021	S-GQDs@@Au-CNS	Biosensor	**Glioma cells** (not specified)	-	A manufactured biosensor allows for the detection of low concentrations of glioblastoma cells, starting with 40 cells per mL.
Perini et al. [35]	2022	GQD	Pretreatment	**U87MG** (200 μg/mL)	-	At 200 µg/mL, GQD + 1 µM Dox reduces U87 spheroid viability by 30%, while adding NIR irradiation further decreases spheroid volume by 70% over 14 days. However, NIR irradiation does not enhance viability reduction beyond GQD + Dox treatment alone.
Perini et al. [36]	2023	GQD	PTT/Pretreatment	**U87**, human fibroblasts (50; 100; 200; 250 μg/mL)	-	GQD (50–250 µg/mL) is non-cytotoxic and enhances membrane fluidity at 250 µg/mL in tested cells. When combined with 1 µM Dox, it increases Dox uptake and reduces viability at ≥200 µg/mL in U87 cells. A similar effect occurs with GQD at 200–250 µg/mL alongside 100 µM TMZ. In 3D U87 spheroids, GQD alone or with drugs does not affect viability but increases membrane permeability at 200 µg/mL. The combination of GQD and PTT reduces spheroid growth, with PTT plus chemotherapy being more effective than the drugs alone. This approach also improves drug penetration, boosts immune cell recruitment, and elevates ROS production in treated spheroids.
Ostovar et al. [37]	2023	CS/CMC/GQD/Zn@QUR	Drug delivery	**U87**, L929	-	The composite CS/CMC/GQD/Zn@QUR has an improved controlled release profile and shows greater inhibitory effects on U87 cells compared to pure QUR, while keeping toxicity levels acceptable in non-cancerous cells.
Wang et al. [38]	2025	Ang-2/GQD-NPG/GCE	Biosensor	**U251**, 3T3, SCC7, 4T1, B16–F10, HeLa, HepG2, **LN229**, PC-9, LL/2, hCMEC/D3 and LO2	-	Ang-2/GQD-NPG/GCE enables selective glioblastoma cell detection in serum and culture, with a detection limit of 1 cell/mL and stability for 21 days.

## Data Availability

No new data were created or analyzed in this study.

## Abbreviations

The following abbreviations are used in this manuscript:

DLS	Digital Light Scattering
AFM	Atomic Force Microscopy
TEM	Transmission Electron Microscopy
TNF-α	Tumor Necrosis Factor alpha
IL-6	Interleukin 6
IL-10	Interleukin 10
BDNF	Brain-Derived Neurotropic Factor
N.D.	No Data
